# A controlled study on the diagnostic accuracy of panoramic and peri-apical radiography for detecting furcation involvement

**DOI:** 10.1186/s12903-021-01460-z

**Published:** 2021-03-12

**Authors:** Gijs Berghuis, Jan Cosyn, Hugo De Bruyn, Geert Hommez, Melissa Dierens, Véronique Christiaens

**Affiliations:** 1 Oral Health Sciences, Department of Periodontology and Oral Implantology, Faculty of Medicine and Health Sciences, Ghent University, Corneel Heymanslaan 10, 9000 Ghent, Belgium; 2 Oral Health Research Group (ORHE), Faculty of Medicine and Pharmacy, Vrije Universiteit Brussel (VUB), Laarbeeklaan 103, 1090 Brussels, Belgium; 3 Department of Dentistry- Implantology and Periodontology, Research Institute Health Sciences, Radboud University Medical Center, Philips Van Leydenlaan 25, 6525 EX Nijmegen, The Netherlands

**Keywords:** Diagnosis, Furcation involvement, Periodontal disease, CBCT, Panoramic radiography, Intra-oral radiography

## Abstract

**Background:**

The aims of this study were (1) to determine the accuracy, sensitivity, and specificity of panoramic and peri-apical radiographs in diagnosing furcation involvement, as well as (2) to evaluate the possible impact of clinical experience on these diagnostic parameters.

**Methods:**

An existing radiographic dataset of periodontitis patients requiring implant surgery was retrospectively examined for furcation involvement. Criteria for inclusion were the presence of a CBCT, panoramic and peri-apical radiograph of the site of interest within a one-year time frame. All furcation sites were classified using the CBCT, which was considered as the gold standard, according to Hamp’s index (1975). Ten experienced examiners and 10 trainees were asked to assess furcation involvement for the same defects using only the corresponding panoramic and peri-apical radiographs. Absolute agreement, Cohen’s weighted kappa, sensitivity, specificity and ROC-curves were analyzed.

**Results:**

The study sample included 60 furcation sites in 29 multi-rooted teeth from 17 patients. On average, 20/60 furcations were correctly classified according to the panoramic radiographs, corresponding to a weighted kappa score of 0.209, indicating slight agreement. Similarly, an average of 19/60 furcations were correctly classified according to the peri-apical radiographs, corresponding to a weighted kappa score of 0.211, also indicating slight agreement. No significant difference between panoramic and peri-apical radiography was found (*P* = 0.903). When recategorizing FI Grades into ‘no to limited FI’ (FI Grade 0 and I) and ‘advanced FI’ (FI Grade II and III), the panoramic and peri-apical radiography showed low sensitivity (0.558 and 0.441, respectively), yet high specificity (0.791 and 0.790, respectively) for identifying advanced FI. The ROC-curves for the panoramic and peri-apical radiographs were 0.79 and 0.69 respectively. No significant difference was found between experienced periodontists and trainees (*P* = 0.257 versus *P* = 0.880).

**Conclusion:**

Panoramic and peri-apical radiography are relevant tools in the diagnosis of FI and provide high specificity. Ideally, they are best used in combination with furcation probing, which shows high sensitivity. Furthermore, clinical experience does not seem to improve the accuracy of a radiological diagnosis of furcation sites.

***Trial registration*:**

Patient radiographic datasets were retrospectively analyzed.

## Background

The diagnosis of periodontitis is based on clinical as well as radiographic examination [1, 2]. Two-dimensional radiographic examination by means of peri-apical radiography, is still the standard method for assessing marginal bone loss [3]. In addition, decay, root morphology and resorptions can be identified [4]. Ideally, peri-apical radiographs should be taken using the paralleling technique to optimize the diagnostic quality [5].

Panoramic radiographs may occasionally be combined with peri-apical radiographs as an alternative to a full-mouth series of peri-apical radiographs to reduce the total radiation dose [6]. Unfortunately, there is a notable variation in the selection and use of appropriate radiographic methods to assess periodontal diseases in general dental practice [7].

A detailed diagnosis of periodontitis is more challenging in the posterior dentition because of furcation and root proximities. In the case of periodontal attachment loss around multi-rooted teeth, bone resorbs stepwise and the furcation may become involved [8]. Such furcation involvement (FI) is a clinical parameter and determines the severity of pathological resorption of the supporting alveolar bone within a furcation [9]. Interradicular osseous defects are associated with loss of tooth support. Furthermore, there is an association that ecological niches are situated in these interradicular defects. Ecological niches could be locus-specific risk factors [10] and induce further progression of pathological bone breakdown by means of apical downgrowth and spread of subgingival plaque between the root cones [4]. First, a widening of the periodontal space in combination with inflammatory and cellular fluid exudation is observed, followed by epithelial proliferation into the access of the furcation [11].

The degree of attachment loss in the furcation of multi-rooted teeth may be categorized into four grades based on horizontal measurements as described by Hamp et al. [12]. Grade 0 implies that the furcation is not accessible with a curved Nabers furcation probe. Grade Ι represents horizontal attachment loss up to 3 mm, while Grade ΙΙ represents horizontal attachment loss exceeding 3 mm, but no detectable “through and through destruction”. Grade III represents horizontal “through-and-through” destruction of the periodontal tissues in the furcation. Vertical defects associated with intra-bony pockets could also be considered as a pattern of bone destruction [11]. Given the variation of molar root anatomy in the mandible versus the maxilla as well as variations in the morphology of multi-rooted teeth, it is extremely challenging to exactly identify the stage of periodontal breakdown.

In treatment planning, FI is an important criterion to consider when making a prognosis for a tooth being possibly secure, doubtful, or irrational to treat. This is based on the fact that FI is considered a locus minoris resistentiae increasing the risk for tooth loss. In this respect, McGuire and Nunn [13] reported that a tooth with Grade III FI has an increased risk of being irrational to treat compared to a tooth with FI Grade II, Grade I, or no FI. Multirooted teeth with any FI are at greater risk for further attachment loss, thereby compromising the long-term prognosis [14]. Given this consideration, it follows that FI should be considered in the decision-making process together along with other dental- and patient-related factors. Furthermore, the reduced accessibility and complicated anatomical architecture of furcation defects limits the efficacy of initial periodontal therapy [15, 16]. When residual pockets ≥ 6 mm and full-mouth bleeding scores < 30% persist, additional periodontal surgery may be necessary [17].

Cone beam computed tomography (CBCT) is a relatively new 3D approach in oral imaging [8, 18]. CBCT provides reconstructions of dental anatomy and pathology in axial, coronal and sagittal planes making a detailed and accurate diagnosis possible [19]. The FI extent can be easily identified as CBCT is a 3D digital reconstruction of a complex clinical situation, whereas panoramic and peri-apical radiographs are limited to a 2D perspective [8]. For a medium field of view (FOV) CBCT scan, the radiation dose ranges from 9 to 560 μSv, which is much lower than the effective radiation dose of a conventional Computed Tomography (CT) scan [20]. The effective radiation dose for a panoramic radiograph is 3–24.3 μSv and 34.9–104.71 μSv for an intra-oral full-mouth series of peri-apical radiographs [21, 22].

Two-dimensional radiographic examination is limited in assessing marginal bone levels and incipient inter-radicular bone loss because of overlap of bone and surrounding anatomic structures [8]. Underestimation of alveolar bone loss ranges from 13 to 32% in panoramic radiography versus 9–21% in peri-apical radiography [23]. However, peri-apical radiography is more accurate in detecting and assessing the dimensions of periodontal bone defects [24]. Unfortunately, detailed data on the accuracy of panoramic and peri-apical radiography for the detection of FI has not been published. Hence, the primary objective of this controlled study was to determine diagnostic accuracy, sensitivity (SENS) and specificity (SPEC) of panoramic and peri-apical radiography for detecting furcation involvement [1] and to evaluate the possible impact of clinical experience on these diagnostic parameters [2].

## Methods

### Patient and site selection

Patient demographics and radiographs were retrospectively selected from Ghent University Hospital records of patients who had been treated at the Department of Periodontology and Oral Implantology. Patients demonstrated varying severity and extent of periodontal disease, had at least one molar in situ without merged roots and had one or more implants placed following periodontal therapy. Given that, CBCTs were available from all patients. Additional inclusion criteria were the presence of a panoramic and peri-apical radiograph of the site of interest within a one-year time frame. The study was conducted in accordance with the Helsinki declaration of 1975 as revised in 2000. The protocol was approved by the ethical committee of the Ghent University Hospital (B670201523577). Consent to participate was not applicable for all individual participants included in the study.

### Examiners

Ten staff members who had been working for at least 5 years as a periodontist and 10 postgraduate trainees in Periodontology and Oral Implantology participated in the evaluation of the radiographs. As it was our intention to assess the diagnostic value of panoramic and peri-apical radiograph for assessing advanced FI in daily practice, the clinicians were deliberately not trained and calibrated beforehand.

### Assessment of FI on the basis of cone beam computed tomography (CBCT), panoramic and peri-apical radiographs

For every furcation site the degree of horizontal alveolar bone loss was established on CBCT (Planmeca ProMax® 3D Max, Helsinki, Finland) by two experienced clinicians (VC, MD). The CBCTs were taken with a standardized protocol for three-dimensional implant planning (⌀100 × 55 mm or ⌀100 × 90 mm medium FOV with 200 µm voxel size, 90 kV tube voltage and 12 s scanning time). Mean mA was 5.6 and ranged between 4.5 and 7.1 depending on the skull size and weight of the patient. All CBCT volumes were available in DICOM format, analyzed with Planmeca Romexis© software (Romexis 4.5.2.R, Helsinki, Finland) and carefully assessed in all different planes on a 24-inch monitor (Barco Eonis® MDRC-2224 BL, Kortrijk, Belgium) with dimmed surrounded light. The data on horizontal alveolar bone loss were transformed to the Hamp et al. [12] classification (= gold standard). Whenever a different Grade was found among the two experienced clinicians, the final Grade was determined following discussion.

Every clinician scored the same sites using the Hamp et al. [12] classification on the basis of panoramic (Planmeca ProMax dimax4 2DScara3+Pan+Cephalostat GUI version 3.7.1.0.r, Helsinki, Finland) and peri-apical radiographs (Dürr Dental Vistascan Phosphor plates size 2+, Bietichheim-Bissingen, Germany) under optimal conditions: separately and in a quiet semi-dark room with dimmed surrounded light. A washout period of one month between measuring the panoramic and peri-apical radiographs was respected to eliminate possible effects from previous measurements.

Panoramic images had been taken with 66 kV tube voltage and 8 mA. Digital peri-apical radiographs had been taken with Rinn XCP® Paralleling holders (Dentsply®, Weybridge, UK). The active exposure time ranged between 0.08 and 0.12 s with 70 kV tube voltage and 7 mA. Peri-apical radiographs were developed with an image plate scanner (Dürr Dental VistaScan Mini Plus, Bietichheim-Bissingen, Germany).

All two-dimensional radiographic images were viewed in full-screen modus with specialized imaging software (Mediadent, Image Level version 6.14.4.24F, Kruibeke, Belgium) on a 24-inch monitor (Barco Eonis® MDRC-2224 BL, Kortrijk, Belgium).

### Statistical analysis

Data analysis was performed in IBM SPSS® Statistics 25 with the furcation site as the statistical unit. Descriptive statistics included frequency distributions for categorical variables (gender, FI Grade) and mean values and standard deviations for continuous variables (age). The FI Grade as registered on CBCT was considered the true FI Grade (= gold standard). Absolute agreement and Cohen’s weighted kappa was calculated to determine the accuracy of panoramic and peri-apical radiography in assessing the FI Grade. FI Grades were recategorized into ‘no to limited FI’ (FI Grade 0 and I) and ‘advanced FI’ (FI Grade II and III) in order to determine the diagnostic sensitivity (SENS), specificity (SPEC), positive predictive value (PPV) and negative predictive value (NPV) of panoramic and peri-apical radiography in identifying advanced FI. For all diagnostic parameters 95% confidence intervals were calculated. To explore the possible impact of clinical experience, diagnostic parameters and area under ROC-curves were compared between experienced periodontists and postgraduate trainees using the Mann Whitney U-test. Finally, composite Receiver Operating Characteristic (ROC)-curves [25] were constructed with Python™ Software 3.8.0 to assess the diagnostic value of both imaging techniques on the basis of pooled data of all examiners, experienced clinicians or trainees. Therefore, the state value (CBCT) was recategorized into ‘no to limited FI’ (FI Grade 0 and I) and ‘advanced FI’ (FI Grade II and III). Test values included all FI Grades. The level of significance was set at 0.05.

## Results

### Patient and site selection

In total, 60 furcation sites of 29 multi-rooted teeth from 17 patients (6 males, 10 females; mean age 62) could be included in this controlled clinical study. The original sample included 75 furcation sites, yet FI could not be assessed for 15 sites due to restoration materials causing CBCT artifacts. Fourteen of the 29 teeth were maxillary molars. Of those, 5 were first molars, 8 s molars and 1 third molar. Fifteen of the 29 teeth were mandibular molars. Of those, 5 were first molars, 8 s molars and 2 third molars. Twelve of the included furcation sites were mesiopalatal, 24 were buccal, 12 were distopalatal and 12 were lingual sites.

### Examiners

Periodontists had a mean age of 43 years (SD 10.1) and included 6 males and 4 females. They had on average 16 years (SD 9.6) of clinical experience in private practice. Trainees had a mean age of 29 years (SD 1.9) and included 9 males and 1 female.

### Assessment of FI on the basis of cone beam computed tomography (CBCT), panoramic and peri-apical radiographs

#### CBCT as gold standard

Based on detailed CBCT analysis, the degree of horizontal alveolar bone loss at the level of the furcation was classified as Grade 0 for 19 sites, Grade I for 25 sites, Grade II for 9 sites, Grade III for 7 sites.

#### Panoramic radiography versus CBCT

Details on all diagnostic parameters of panoramic radiography for assessing FI are shown in Table 1. On average 19/60 (SD 4.2) furcation sites were correctly classified on the basis of panoramic radiography. This corresponded to a weighted kappa score of 0.209 (95% CI 0.060–0.376), indicative of slight agreement. On average, 10/19 (SD 4.0) furcation sites with FI Grade 0 were correctly identified. For FI Grade I, II and III, the proportion of correct assessments were 6/25 (SD 2.8), 2/9 (SD 1.0) and 2/7 (SD 2.2), respectively (Table 2). Weighted kappa scores for the experienced clinicians were 0.231 (95% CI 0.052–0.413) and 0.186 (95% CI 0.067–0.338) for the trainees. Weighted kappa scores in the maxilla were 0.301 (95% CI 0.020–0.525) and 0.238 (95% CI 0.030–0.532) in the mandible. There was no significant difference between experienced clinicians and trainees, nor between the maxilla and mandible (*P* > 0.257).

**Table 1:** Diagnostic parameters of panoramic radiography for assessing FI

	Experienced clinicians	Trainees	*P *value	All clinicians
Accuracy	0.231 (95% CI 0.052–0.413) AUC: 0.79	0.186 (95% CI 0.067–0.338) AUC: 0.77	0.257	0.209 (95% CI 0.060–0.376) AUC: 0.79
Sensitivity^a^	0.487 (95% CI 0.421–0.553)	0.624 (95% CI 0.515–0.733)	0.049	0.558 (95% CI 0.490–0.622)
Specificity^a^	0.842 (95% CI 0.799–0.884)	0.740 (95% CI 0.656–0.824)	0.082	0.791 (95% CI 0.742–0.840)
Positive Predictive Value*	0.534 (95% CI 0.436–0.631)	0.487 (95% CI 0.286–0.689)	0.473	0.511 (95% CI 0.409–0.612)
Negative Predictive Value*	0.796 (95% CI 0.727–0.866)	0.811 (95% CI 0.694–0.928)	0.940	0.804 (95% CI 0.742–0.865)

^a^No to limited FI = FI Grade 0 and FI Grade I; Advanced FI = FI Grade II and FI Grade III, AUC = area under (ROC)-curve

**Table 2:** Correctly identified furcation involvements sorted per FI Grade and type of radiography

Examiner	FI Grade 0 (n = 19)		FI Grade I (n = 25)		FI Grade II (n = 9)		FI Grade III (n = 7)	
	Pano	PA	Pano	PA	Pano	PA	Pano	PA
E(1)	10	7	6	1	0	0	5	4
E(2)	9	6	3	2	1	0	7	5
E(3)	11	13	2	4	2	1	1	3
E(4)	10	12	5	2	2	0	5	7
E(5)	13	12	6	8	4	1	0	2
E(6)	13	9	8	14	3	1	0	3
E(7)	11	13	3	4	3	3	2	–
E(8)	8	9	7	9	2	0	2	3
E(9)	13	13	4	2	2	0	1	1
E(10)	9	8	9	8	2	2	5	4
T(1)	13	10	6	10	1	1	0	5
T(2)	4	6	8	6	0	0	2	3
T(3)	11	11	8	3	2	0	2	2
T(4)	14	11	4	8	0	1	–	2
T(5)	1	3	8	2	1	1	0	5
T(6)	18	17	1	2	1	0	–	3
T(7)	10	10	5	4	1	0	2	–
T(8)	6	5	13	9	2	2	5	3
T(9)	8	8	6	8	2	1	2	1
T(10)	4	6	5	3	1	0	0	2
Mean	10	9	6	5	2	1	2	3

*Pano* panoramic radiography, *PA* peri-apical radiography, *E* experienced clinician, *T* trainee

When recategorizing FI into ‘no to limited FI’ (FI Grade 0 and I) and ‘advanced FI’ (FI Grade II and III), SENS of panoramic radiography amounted to 0.558 (95% CI 0.490– 0.622) and SPEC was 0.791 (95% CI 0.742–0.840). The results on PPV and NPV were 0.511 (95% CI 0.409–0.612) and 0.804 (95% CI 0.742–0.865), respectively. SENS was significantly lower for experienced clinicians than for trainees (*P* = 0.049). There was no significant difference between experienced clinicians and trainees for the other diagnostic parameters (*P* ≥ 0.082).

**Fig. 1:**
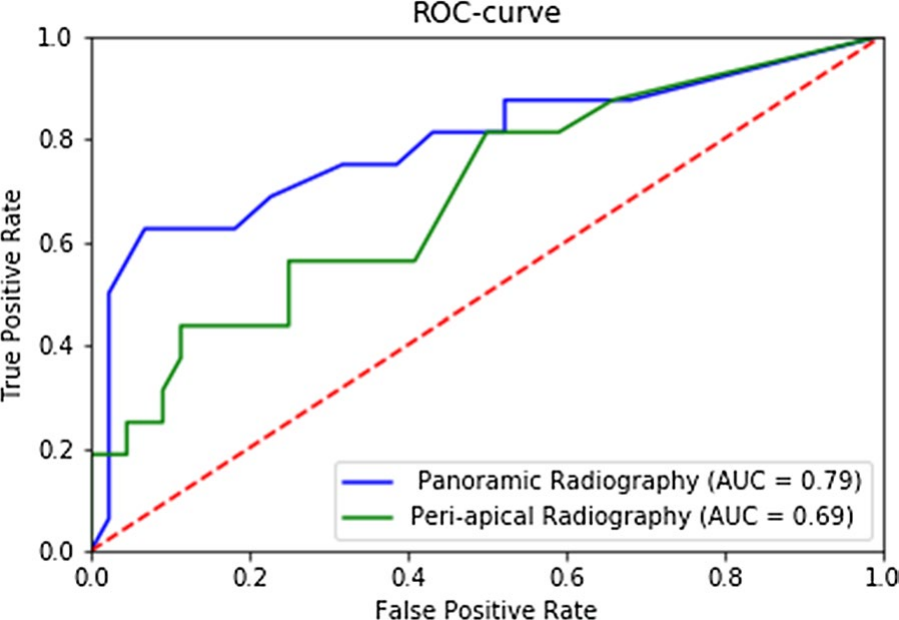
Composite ROC-curve for panoramic versus peri-apical radiography (all examiners)

**Fig. 2:**
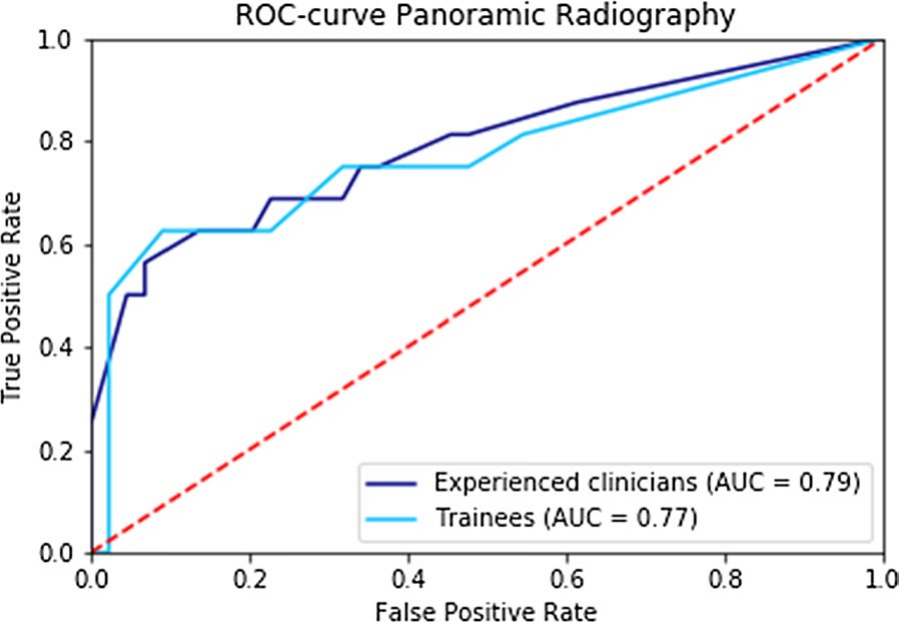
Composite ROC-curve for experienced clinicians versus trainees (panoramic radiography)

Composite ROC analysis showed an area under the curve of 0.79 for all examiners (Fig. 1). For the experienced clinicians the area under the ROC-curve was 0.79 and 0.77 for the trainees (Fig. 2). There was no significant difference between experienced clinicians and trainees (*P* = 0.289).

#### Peri-apical radiography versus CBCT

Details on all diagnostic parameters of peri-apical radiography for assessing FI are shown in Table 3. On average 19/60 (SD 4.2) furcation sites were correctly classified on the basis of peri-apical radiography. This corresponded to a weighted kappa score of 0.211 (95% CI 0.051–0.404), indicative of slight agreement. No significant difference between panoramic and peri-apical radiography was found (*P* = 0.903). On average, 9/19 (SD 3.4) furcation sites with FI Grade 0 were correctly identified. For FI Grade I, II and III, the proportion of correct assessments amounted to 5/25 (SD 3.6), 1/9 (SD 0.9) and 3/7 (SD 1.6), respectively (Table 2). Weighted kappa scores of 0.208 (95% CI 0.047–0.389) were calculated for the experienced clinicians and 0.215 (95% CI 0.055–0.418) for trainees. Weighted kappa scores of 0.257 (95% CI 0.015–0.341) were calculated for the maxilla and 0.151 (95% CI 0.015–0.341) for the mandible. There was no significant difference between experienced clinicians and trainees, nor between the maxilla and mandible (*P* > 0.461).

**Table 3:** Diagnostic parameters of peri-apical radiography for assessing FI

	Experienced clinicians	Trainees	*P*-value	All clinicians
Accuracy	0.208 (95% CI 0.047–0.389) AUC: 0.69	0.215 (95% CI 0.055–0.418) AUC: 0.63	0.880	0.221 (95% CI 0.051–0.404) AUC: 0.69
Sensitivity^a^	0.441 (95% CI 0.373–0.508)	0.442 (95% CI 0.339–0.545)	0.425	0.441 (95% CI 0.386–0.497)
Specificity^a^	0.793 (95% CI 0.758–0.825)	0.788 (95% CI 0.751–0.825)	0.820	0.790 (95% CI 0.768–0.812)
Positive predictive value^a^	0.454 (95% CI 0.383–0.525)	0.424 (95% CI 0.313–0.535)	0.677	0.439 (95% CI 0.413–0.846)
Negative predictive value^a^	0.783 (95% CI 0.683–0.884)	0.776 (95% CI 0.667–0.885)	0.596	0.780 (95% CI 0.748–0.835)

^a^No to limited FI = FI Grade 0 and FI Grade I; Advanced FI = FI Grade II and FI Grade III, AUC = Area Under (ROC)-Curve

When recategorizing FI into ‘no to limited FI’ (FI Grade 0 and I) and ‘advanced FI’ (FI Grade II and III), SENS of peri-apical radiography was 0.441 (95% CI 0.386–0.497), while SPEC was 0.790 (95% CI 0.768–0.812). The results on PPV and NPV were 0.439 (95% CI 0.413–0.846) and 0.780 (95% CI 0.748–0.835), respectively. There was no significant difference between experienced clinicians and trainees for any of the diagnostic parameters (*P* ≥ 0.425).

**Fig. 3:**
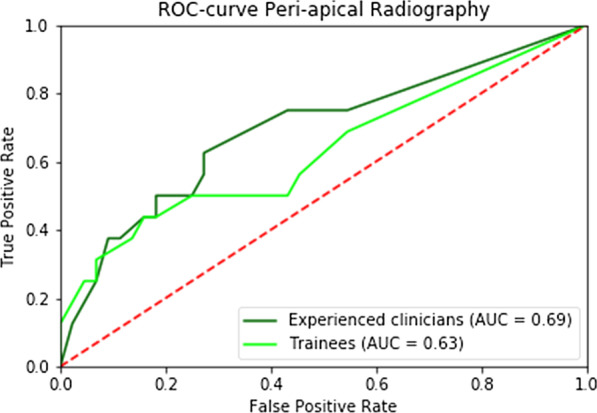
Composite ROC-curve for experienced clinicians versus trainees (peri-apical radiography)

**Fig. 4:**
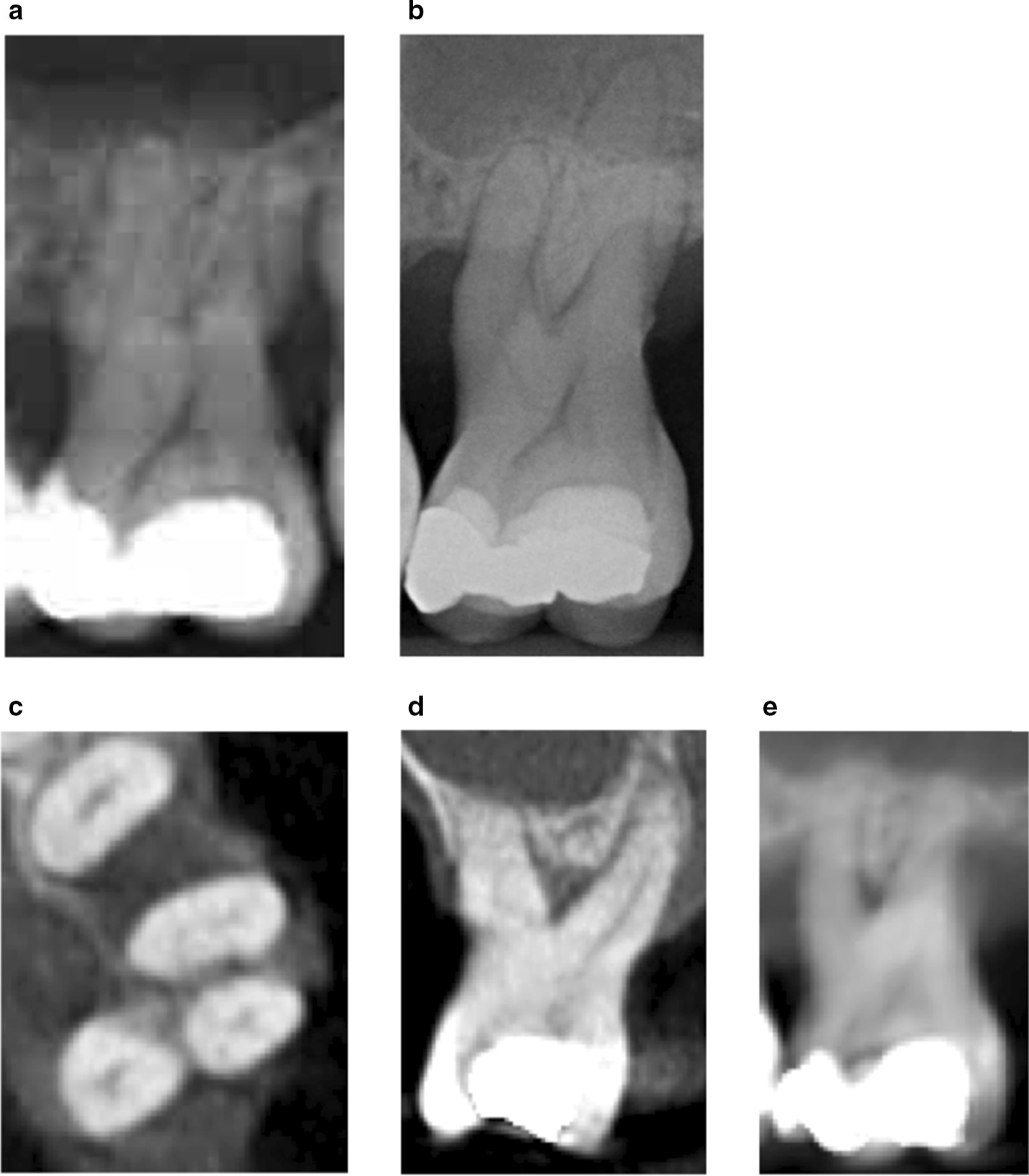
Clinical example of a maxillary molar with FI Grade III at the mesiopalatal site. **a** Enlarged panoramic radiograph, **b** peri-apical radiograph, **c** CBCT: axial slide, **d** CBCT: coronal slide, **e** CBCT: sagittal slide

Composite ROC analysis showed an area under the curve of 0.69 for all examiners (Fig. 1). For the experienced clinicians the area was 0.69 and for trainees it was 0.63 (Fig. 3). There was no significant difference between experienced clinicians and trainees (*P* = 0.759). Figure 4 shows a clinical example of a maxillary molar with FI Grade III.

## Discussion

The main objective of this study was to compare the accuracy of panoramic and peri-apical radiography with CBCT in detecting FI. Since panoramic and peri-apical radiographs are still taken regularly in daily general dental practice, data on their accuracy is of pivotal relevance [26]. Proper assessment of FI with a high degree of accuracy is also important for adequate treatment management of periodontitis patients [11].

FI is considered a locus minoris resistentiae in periodontal disease progression with high prevalence in periodontitis patients. Svärdström and Wennstrom [27] studied the distribution of furcation lesions and found that these lesions were most prevalent in the maxilla and more specific at distal sites of upper first molars. Furcation lesions could be found in more than 50% of chronic periodontitis patients older than 30 years. Every second molar was involved in patients older than 40 years.

Measuring clinical attachment level by horizontal probing is a standard tool in clinical periodontology. However, access to furcations is not always easy and furcations may be difficult to predict in architecture, number of walls and extent. An ideal method to identify FI is by reflecting a mucoperiosteal flap. Evidently, this is impossible for ethical reasons when there is no pathology or clear clinical indication for surgery. Intra-surgical registrations have been often used as a gold standard in the diagnosis of periodontal defects indicated for surgery [23, 26, 28]. CBCT was used as gold standard in the present study since these were available in the context of planning implant surgery. CBCT has been shown to be a good gold standard in multiple studies describing high levels of agreement between pre-CBCT FIs and intra-surgical findings [29, 30]. In addition, CBCT enables to visualize root characteristics such as fusions and proximities from multiple perspectives.

These findings explains that inter-rater reliability is insufficient to conclude that one radiographic method is superior towards another for finding FI compared with CBCT. On the other hand, all diagnostic parameters need to be evaluated in detail before clinical recommendations can be made. Diagnostic parameters such as SENS, SPEC, PPV and NPV can only be calculated for dichotomous variables. For this purpose, FI Grades were recategorized into ‘no to limited FI’ (FI Grade 0 and I) and ‘advanced FI’ (FI Grade II and III). This recategorization makes sense from a clinical point of view since FI Grade 0 and I require no or limited non-surgical therapy, whereas FI Grade II and III need advanced surgical intervention. Panoramic and peri-apical radiography showed low SENS (0.550 and 0.441, respectively), yet high SPEC (0.791 and 0.790, respectively) for identifying advanced FI. These findings imply relatively high false negative ratings and low false positive ratings. In other words, advanced FI is frequently overlooked, but when it is identified on the basis of panoramic radiography or peri-apical radiography, it is most likely present. This can be explained by the fact that panoramic and peri-apical radiographs are two-dimensional images of a three-dimensional anatomy, hence a certain overlap is not unexpected. Even advanced lesions may be masked due to superimposition of bone, roots and restorative materials. On the other hand, peri-apical radiographs score superiorly for image quality (brightness, contrast) and bone details (quality of bone, contour of lamina dura) [31, 32]. Panoramic radiographs provide a good overview, yet image distortion is an important limitation [6, 33]. Interestingly however, is that panoramic radiography was not inferior to peri-apical radiography for detecting FI in this study. This may be explained by the fact that the quality of digital panoramic radiographs has improved dramatically in recent years. ROC-curves showed similar results with a slightly higher area under the curve for panoramic radiography when compared to peri-apical radiography (0.79 versus 0.69).

The PPV and NPV results are more difficult to interpret than SENS and SPEC since both are affected by the prevalence of advanced FI within the study sample. In this study, 16/60 furcations were Grade II or III. Only when this proportion resembles the proportion of advanced FI in the population, the data on PPV and NPV are valid. Clearly, the present study was based on a convenient sample of patients seeking implant therapy, which may not adequately represent the population. In addition, substantial regional differences may exist in the prevalence of advanced FI among periodontitis patients.

In a retrospective study of Darby et al. [11], the diagnostic accuracy of furcation probing for detecting FI was investigated using CBCT as gold standard. Only 22% of the furcation sites were clinically accurate in grading compared with CBCT and 58% were overestimated. These findings indicate high SENS and low SPEC of furcation probing. Accuracy of furcation probing is dependent on factors such as inclination and angulation of the probe, variability in operator’s technique/ inherent probing error, amount of force used when probing, tooth position, presence of adjacent teeth, restricted visualization of the probe due to limited mouth opening and difficult access to the entrance of the furcation. Also, the clinician can accidentally score the furcation concavity than the furcation itself, as deep root concavities may be confused with FI. All these factors may explain the high overestimation of clinical FI measurements. On the basis of the results of Darby et al. [11] and the results of the present study, it is clear that neither furcation probing, nor panoramic/peri-apical radiography are excellent examination methods on their own for the detection of FI. However, when these methods are combined more furcations may be accurately assessed given the fact that furcation probing demonstrates high SENS whereas panoramic/peri-apical radiography show high SPEC. This is in line with a study of Gusmao et al. [34] and Greatz et al. [35] indicating that both furcation probing and radiographical assessment should be used in situations of suspected FI.

When making surgical decisions, it seems particularly attractive to take a three-dimensional radiograph [8]. Indeed, when teeth require complex periodontal therapy as well as restorative treatment, CBCT might be a good additional tool for an accurate assessment and prognosis of multi-rooted teeth. Inaccurate diagnosis can lead to irreversible treatment planning decisions [11]. Still, the diagnostic benefits of CBCT must be carefully balanced against a higher radiation dose [35]. The radiation dose of a low-dose small-field CBCT is slightly higher when compared to a digital panoramic radiograph, yet substantially higher when compared to a peri-apical radiograph [11]. When translated to clinical practice, a low-dose small-field CBCT might be considered when the architecture of the defect has a clear impact on the treatment strategy [36]. This may apply to mandibular furcations with FI Grade II since these qualify for regenerative periodontal surgery.

An interesting finding was that diagnostic parameters for panoramic and peri-apical radiography were not affected by the experience of the clinician. Hence, gaining clinical experience does not seem to improve the accuracy of a radiological diagnosis of furcation sites.

When interpreting the results of the present study, several limitations should be taken into account. First, this is a retrospective study based on a convenient sample. Second, we used the most common classification on FI because of its simplicity. Furthermore, FI also has a vertical component [37], which was not considered here. By classifying the vertical component, it could clinically influence the treatment strategy. Essentially, treatment of FI may involve periodontal regeneration, root resection, amputation, tunneling techniques and tooth extraction [38–42]. Prognostically, the vertical component increases the risk of tooth loss significantly [43] and needs to be regarded as a risk factor in personalized maintenance programs [44]. Third, CBCT was used as gold standard because of ethical restrictions. Assessing FI by means of intra-surgical examination remains the most accurate method [11]. As there was no significant difference found between panoramic and peri-apical radiography, the principle of justification with a need for patient-specific imaging should always be kept in mind. Additionally, decision making should be made on a case-by-case basis according to the ALARA principle.

## Conclusions

In conclusion, panoramic and peri-apical radiography are relevant in the diagnosis of FI given high specificity. However, when clinical examination indicates pathology, diagnostic accuracy may be improved when clinical measurements are combined with radiographic examination. In clinical applications where CBCT images are present for justified reasons, the available CBCT images are more accurate than 2D images to assess furcation involvement. These are preferably combined with furcation probing, which shows high sensitivity. Clinical experience does not seem to improve the accuracy of a radiological diagnosis of furcation sites.

## Data Availability

The study was supported by the authors and their institutions. Patient demographics and radiographs were retrospectively selected from Ghent University Hospital records of patients who had been treated at the Department of Periodontology and Oral Implantology. All data generated or analysed during this study are included in this published article and its supplementary information files.

## Abbreviations

SPEC: Specificity
SENS: Sensitivity
PPV: Positive predictive value
NVP: Negative predictive value
ROC: Receiver operating characteristic
SD: Standard deviation
FI: Furcation involvement
CBCT: Cone-beam computer tomography
FOV: Field of view
